# A figurational approach to soft power and sport events. The case of the FIFA World Cup Qatar 2022™

**DOI:** 10.3389/fspor.2023.1142878

**Published:** 2023-04-06

**Authors:** Hans Erik Næss

**Affiliations:** Department of Leadership and Organization, Kristiania University College, Oslo, Norway

**Keywords:** mega-events, geopolitics, relational sociology, historicism, scale

## Abstract

Soft power' is a term often used to explain how states seek to be appealing and attractive to others to increase their geopolitical influence instead of using military force or economic threats. As part of a soft power agenda, sport events showcase the values, culture and imagery of a host nation, and through that, ideally, attract investors, tourists and attention. But there are problems with the concept, especially when it is adopted by sport events where it is used as a metaphor, as a heuristic device, and a descriptor of current affairs, to name a few. In particular, the concept does not necessarily capture the accumulation of tit-for-tat strategies and bargaining in geopolitics where sport events are involved. In contrast, by using the FIFA World Cup Qatar 2022™ as key example and drawing upon Norbert Elias' figurational sociology as source of theoretical refinement, this article discusses how soft power can become more precise as an analytical category in a context of sport and geopolitics.

## Introduction

This aim of this article is to refine “soft power” as an analytical category in connection with major sport events and geopolitics. The need for conceptual refinement has grown due to the large number of studies of the way in which sport events are used to symbolize the attractive values and imagery of a host nation, in both emerging and developed states, for internal and external soft power purposes. Sport events have become media-driven, commercial and strategic mechanisms through which states may channel “brand-building” exercises domestically and/or in the geopolitical arena (1–9).

Despite the increasing use of the concept and its differentiation by researchers in various disciplines, the bargaining of political capital in international relations where sport events are involved demands further investigations of how to respecify “soft power” as analytical category, that is, a label which through its conceptual features, aggregates elements of a phenomenon and enables studies of it in practice. For that same reason, others have made considerable progress in reconceptualizing soft power in general. In addition to Nye's own work on this (10, 11), Bakalov (12), for example, also uses the history of European integration to address unresolved problems with the concept and to offer a comprehensive update located in the “contentious politics research programme”. Yet, as I will return to below, there are circumstances unique to sport events which require a more context-sensitive approach. Similar to Bakalov's study (12), the aim is thus not to dismiss the idea of soft power altogether, or to reach a final conclusion of how power works. The purpose is to contribute to the term's operational usefulness for studies of sport events by marrying it with the figurational sociology of Norbert Elias, which to this author's knowledge, is unexplored terrain in social science.

This article starts with three weaknesses in soft power conceptualizations where a merger with Eliasian sociology might rejuvenate the term. First, applications of soft power to contemporary sport events should re-estimate the weight of prior developments (13) because of the influence accumulated by “old” relations and political institutions (14) in geopolitics, that is, “relations between the conduct of a politics of power oriented toward the international level and the geographic frame in which it is carried out” (15; cited and translated by 16). Second, due to the need for “a more nuanced understanding of power relations in world politics” (12), we should explore how soft power depends on the actor's placement in geopolitical networks (such as G7 countries or the Gulf Cooperation Council). Third, we should account for how globalization processes, in a context of soft power and sport, affect the ways mega-events may differ in importance among different group, ie, how these events “are *given scale*—inscribed with size and importance—by the relations actors make in various practices and situations” (17).

Given this context, which will be elaborated further below, this article argues that theoretical refinement may reinforce soft power as analytical category in studies of geopolitics and sport events by addressing these three weaknesses. Drawing upon state-of-the-art research, think-thank reports, official FIFA documents, and media reports to flesh out the analysis it proceeds with a section reviewing the characteristics of soft power. Next, Elias' figurational sociology will be outlined with power as a central theme. The reason why Elias is chosen as representative for a relational approach rather than other relational scholars is because his theories capture the essence of the resource exchange dynamic that is characteristic of soft power tactics through sport. In the following three sections, the Eliasian dimensions which soft power needs to consider in sporting contexts—the stock of influence, the relational position, and the scale of actions—will be discussed in the light of the 2022 FIFA World Cup in Qatar. Finally, for the sake of future research, a conceptual model for using soft power as analytical category is introduced.

## Soft power revisited

The conceptual history of “soft power” is well documented. Yet, because of this history, it is necessary to outline the understanding of it as used in this article. The term was coined by American scholar Joseph S Nye (18, 19) to envision a way for the US to exercise primacy in the globalization age. The focus was on changing the policy of the state from force to attraction and on how an instrumental use of the more attractive aspects of the US in international relations could make its power (more) legitimate, cheaper and more effective. In a post-Cold War landscape, this concept was taken as key to increase its international influence in a new and multi-polar world (20). Revising the construct of power in an institutionalist direction, which according to Bakalov (21) was one of Nye's key theoretical motives, meant seeing it as “instances of A achieving desired outcomes in concert with B” (21), rather than the realist perspective where A controls or dominates B. Nye argued that getting others “to want what you want” (19) was better suited to the new world order than hard power (military and economic force) only. That does not mean shifting from hard to soft power entirely. In fact, Nye argues, “I also said that soft power was only one component of power, and rarely sufficient by itself. The ability to combine hard and soft power into successful strategies where they reinforce each other could be considered “smart power”” (22).

To flesh out soft power, Nye emphasized “intangible power resources” (19), a country's “culture (in places where it is attractive to others), its political values (when it lives up to them at home and abroad), and its foreign policies (when they are seen as legitimate and having moral authority)” (23). These resources should then be packaged to be attractive to whole nations, not just their governments, and promoted through three dimensions of public diplomacy—daily communication with the media and other opinion-makers; strategic communication, or the equivalent of a nation-branding advertising campaign; and lastly, “the development of lasting relationships with key individuals over many years through scholarships, exchanges, training, seminars, conferences, and access to media channels” (24). Whereas public diplomacy contains several “tools of persuasion” (25), four of them are visible in a sporting context: image-building, which entails a nation's self-promotion, such as with the 1994 Lillehammer Winter Olympics as the symbol of so-called Norwegian values (peace, modesty, decorum, and eco-friendliness) the first “green” mega-event; a platform for dialogue, which features the promotion of a relationship, like the 2018 Winter Olympics in Korea, where athletes from North Korea were allowed to compete and march together with South Korean athletes under the Korean Unification Flag in the opening ceremony as a bridge-builder between North and South Korea; trust-building, which may imply being a neutral actor, like the hosting of the Norway Cup, an international youth football cup, which in 1995 brought an Israeli and Palestinian team together for the first time; and reconciliation and integration, as when Mandela used South Africa's hosting of the Rugby World Cup as an attempt to unite the country (25).

Although “soft power” quickly became a catchphrase, it also revived the argument from Keohane and Nye's earlier works that we live in a world characterised by “complex interdependence” (26, 27), that is, “a world in which security and force matter less and countries are connected by multiple social and political relationships” (27). Attention to soft power tactics would give states a relative advantage in this new interdependent world order, a claim that was strengthened by the emergence of the media developments in the 1990s. Amid the information age discourse of the time (28), Keohane and Nye (29) wrote that the development was two-sided. Obviously, the internet enabled anyone with access to be a publisher and NGOs to form networks and mobilize across borders. Media globalization allows the diffusion of information, ideas, artefacts, and other things associated with what is culturally attractive about a country. If credibly diffused, “soft power and free information can, if sufficiently persuasive, change perceptions of self-interest and thereby alter how hard power and strategic information are used” (29). Yet the information revolution, as it is often termed, did not according to Keohane and Nye create a new politics of complex interdependence in the full sense of the term. Information does not exist independently of the already politically structured world and because of the number of non-democratic states, other forms of power remain highly significant (29). Therefore, the interaction between soft and hard power remains crucial.

About twenty years, later there were numerous empirical explorations of whether soft power matters. Using data from 2000 to 2012, one study commissioned by the British Council split “soft power” into categories like cultural institutions, prosperity and internet connectivity, democracy, and foreign aid. Findings showed that the impact of a high culture rank was found to be greater than any of the factors in the models presented for UN voting—including the hard power of a state's economic strength as measured in GDP (30). On the other hand, the construct validity of “soft power” can be questioned because of its conceptual flexibility and operational vagueness, and, in addition, because it has evolved over time (31, 12). While early conceptualizations focused on categorization of power types and instruments for utilizing them, the more recent approaches combine hard and soft power and emphasize the interaction of power types in foreign affairs (17, 32). Apart from the debate on whether soft power relates to resources or behaviour (12), which is addressed for example in the context of the information age (33), some reviews also emphasize that the concept may be less relevant after the terror attacks on the US on September 11, 2001 (34) or because it does not comprehend contemporary geopolitics (35). From an anthropological view, the idea of “culture” is furthermore one of the weaker elements of soft power. Nye (24) underlines that exporting “Hollywood films full of nudity and violence to conservative Muslim countries may produce repulsion rather than soft power” (*p*. 95). But neither Hollywood nor Islam represent entire countries or necessarily form the basis of their people's view of each other. In the case of China, for example, Hubbert (36) reveals that state-sponsored cultural diplomacy programs through the Confucius Institutes fell through because many participants preferred “the real China”.

Despite the findings of the British Council study as well as the usage of soft power in sport for morally legitimate reasons, it is also used for a variety of purposes which are more questionable. Soft power instruments can be confrontational (25), can blend with propaganda, be used to denigrate the legitimacy of others, or be part of “information warfare”. Information overload can furthermore lead to credibility issues for soft power tacticians (29). The state as soft power agent in connection with sport events is moreover not straightforward because of various conceptualizations of “the state”. An example is Koch's (6) study of the 2016 cycling world championship in Qatar, where Grix and Kramareva (5) are criticized for a statist conception of those involved: “when specific actors or decision-makers get subsumed through a nebulous reference to the “state” as an actor, this effectively erases their agency, as well as their unique positionality and specific political agendas” (6). The limits of soft power as a standalone concept are also evident, as Nye puts it: “Creating a Confucius Institute to teach Chinese culture in Manila will not generate attraction if Chinese naval vessels are chasing Philippine fishing boats out of Scarborough Shoal that lies within 200 miles of its coastline” (22). In a study of China, globalization and the 2008 Beijing Olympics, Giulianotti (37) furthermore speak of soft “disempowerment”, the drawback of soft power tactics being seen as fraudulent and causing stronger reactions than it otherwise would have done. Usage of the concept thus requires us to examine questions concerning to whom soft power instruments are applied, to what end, and through which mechanisms. Whereas others have focused on international relations in their attempts to address conceptual issues (12, 21), this article draws on the figurational sociology of Norbert Elias to revise soft power in the context of sport. The next section reviews Elias' theoretization of power and figurations, before the article turns to how this theoretization bears on history, relations and scale.

## A figurational approach to power

Elias' sociological outlook is characterized by an emphasis on relations and processes as the key to understanding and explaining social life. This relational foundation for human interaction is explored through mechanisms of interdependence, from which a figuration develops (38), a term which Elias described as “a structure of mutually oriented and dependent people” (39). According to Curry and Dunning (40), an Eliasian perspective therefore implies an alternative to dualistic perspectives such as individual/society or micro/macro:

we are born as a result of our interdependent parents into a structured collectivity or social world—a world of interdependencies or figurations—which we ourselves played no part in forming prior to our birth and which occupies a particular historical geographical position in time and space (40).

The concept of figuration consequently steers us towards a view of social phenomena in three parts: 1) an orientation away from the present and on to questions of “sociogenesis” (structural-historical dimensions of social life) (41), or simply put, how did “this” come to be?; 2) an orientation towards relational questions, for example, in what ways are social actors inter-related?; and 3) an orientation towards what broader chains of interdependence are involved in any given social phenomenon (42).

Exploring the relational conditions of power is one way to unravel, at least historically, what is accidental and what is not (43) in these processes. According to Elias, “the very hub of the figuration process is a fluctuating, tensile equilibrium, a balance of power moving to and fro, inclining first to one side and then to the other” (39). In contrast to Foucault, another relational power theorist whose interpretation “stretches the concept of power to almost to the extreme” (44), Elias underlines that A and B “can be considered separately, but not as being separate” (39). For soft power analysts, this creates a particular challenge because of Elias” rejection of the individual/society dualism (45), partly because societies across the world differ in their understanding of the individual (45). What is more, “the outcomes of complex processes involving the interweaving of the actions of large numbers of people cannot be explained simply in terms of the intentions of individuals” (46).

I will now turn to how this kind of figurational thinking can be transferred to soft power debates. One suggestion of how to unravel Eliasian power dynamics in a context of sport and soft power, is to focus on the categorization of “the Other” in relation to oneself. Tilly (47), who draws upon Elias in his analysis, argues that “the institutionalization of categorical pairs” (*p*. 8) is what creates durable inequality in society. With the means to define societal categories in pairs—social background (working class, bourgeois), gender (male/female), status (insider/outsider) and so on, even to the case of “us” and “them”—comes a reinforcing effect as the boundaries between these categories are more efficient if they “incorporate already well-established forms of inequality” (47). In a geopolitical context, as I will return to below, the categorization of the Others and their relations with terrorist groups is a key issue in Qatar's leverage as diplomatic go-to actor for the US and France in the Gulf region.

Described like this, the outlook mirrors traditional power theories by making someone do that which they did not want to do and centring conflict of interest as the analytical pivot point (49). Yet, Elias sees power as “a question of labile, shifting balances or ratios” which are “not explainable solely by reference to single factors such as Karl Marx's ideas of the ownership of production or Max Weber's ideas of the control of the means of violence” (40). For example, Hobson (50) argues that an Eliasian approach to international relations means states are “not conceived of as self-constituting billiard balls that are locked into head-on conflicts but take on polymorphous figurational properties”, which is visible in a 1981 lecture given by Elias himself:

If you consider the whole pyramid of states formed by humanity, you see that the states are ordered according to the magnitude of their sources of power. Within this hierarchy there is constant movement. The power positions shift. The difficulty is that each movement also affects the balance of power between America and Russia. If, for example, the relationship between Egypt and Libya changes, that also affects the relationship between the great powers (51)

At the same time, the movements induced by soft power do not necessarily change in the same way as with hard power. Often, actors compete on the same ranking criteria in terms of ideological promotion, nation branding and business opportunities by arguing that their country—as evidenced by a mega-sport event—is a more attractive partner than others (49). Although soft power contests might be seen as explicit in sport, where the culture war metaphor has been used to describe the tension between Europe/US, China, the Middle East and Russia (52), the mechanisms are more diverse than with hard power strides. This difference affects the types and content of geopolitical change through sporting events. Given the regional strife about this in the Gulf region, as I will return to later, and the geopolitical scenario with Qatar's complex relations outside the region, figuration explains why “over the long term it is difficult for any one individual or group to “determine history” since their intentions and actions are always likely to be moderated by others on whom they are dependent” (53).

A text which fleshes out this perspective theoretically, and which is used by the above-mentioned Tilly (47), is a book Elias published together with John Scotson (54) about a local community in the UK, *The Established and the Outsiders*. More specifically, they tried to overcome the micro-macro dichotomy by identifying power relations and processes between a group of “outsiders” and the established ones. These groups shared many social, cultural, and economic characteristics, but the established ones had lived there for a longer time. This alone, according to Elias and Scotson, generated a feeling of superiority and group cohesion among the established to the degree that they controlled the community's positions and norms. To identify the degree of cohesion of “established” relative to “outsider” groups, Elias suggested, we need to analyse the figurational “aspect of dominance-subordination relations”, that is, of figurations in which some are dominant, and others subordinate (55). To illustrate how this mechanism goes beyond the UK community, Elias and Scotson write that:

One group can effectively stigmatise another only as long as it is well established in positions of power from which the stigmatised group is excluded. As long as that is the case, the stigma of collective disgrace attached to the outsiders can be made to stick. Unmitigated contempt and one-sided stigmatisation of outsiders without redress, such as the stigmatisation of the untouchables by the higher castes in India, or that of the African slaves or their descendants in America, signals a very uneven balance of power (54).

Despite this, critics claim that the theory of figuration lacks explanatory relevance because of its vague treatment of “epistemological and ontological properties of social reality” (56). To avoid this flaw in the context of established groups and outsiders, Hogenstijn et al. (57) claim that we need to address the time dimension, dependency relations between established and outsider groups, and the “influences of (developments at) different spatial scales on a figuration.” These dimensions overlap to a great deal with the flaws in soft power conceptualizations introduced above and are transferrable to established and outsiders in geopolitics. The group of countries called G7 (US, Japan, France, West Germany, Canada, Italy and the UK) and G20 is one example. Having been a global force for many years since it formed in the 1970s, and representing 50 per cent of global GDP, G7's influence has shrunk ever since, mostly due to the rise of China. Meanwhile, the G20 is too heterogeneous to make effective decisions (58). Therefore, policy analysts from the political think-tank Bruegel proposed the creation of G7+ (58). It would include China and India, exclude Canada, and gather the European states in Euro-zone.

Although this suggestion did not materialize, the composition of groups like the G7 might change again soon with the increasing geopolitical influence of the Gulf countries. Consequently, both soft power as analytical category and the Eliasian concept of figuration could be rejuvenated in the context of sport by a discussion of the stock of influence in each situation, the relative positioning of actors, and the scaling of events. To be even more precise, the following discussion considers how figurations are distinguished by objective and subjective interdependencies (59). The former term may denote institutional integration, while the latter refers to identity processes and the development of a consciousness as community among members of specific groups. In what follows, I will illustrate these with the FIFA World Cup Qatar 2022™ as key example and focus on objective interdependencies.

## The stock of influence

The first aspect is the “stock of influence” affecting the event host's geopolitical situation. According to Banfield (60), who came up with this theory to explain how political actors make decisions, this stock is the accumulated capacity to influence a process, to decide on an outcome, and to assess the costs of getting it your way. This applies to the hosting of mega-events in sport—their use as soft power instruments depends on their geopolitical influence (or lack of such). The controller of this stock (a state, a sport governing body, or a role, like the US President) may either conserve or “spend” it “in order to secure desired outcomes” (61). As proponents of what is named “historicist historical sociology” claim, human development and state formation is “a social process, one in which contingent historical events, dramas and processes are part of broader interrelations, sequences, plots and concatenations which provide a shape—however difficult to discern—within historical development” (43). With increased division of labour, transnational production systems and shifting political alliances, to name a few elements of social change, Elias claimed that “chains of interdependence” (62) have emerged throughout history and with them a certain power dynamic. In a similar vein, Nye (63) refers to Lundestad's term (64) “empire by invitation” when explaining the growth of the US as a superpower after the Second World War: The US was encouraged to become a superpower because of the allies it gained.

As a result, geopolitical influence does not come overnight. In addition, this development of interdependency chains is not always included in studies of the use of sport for soft power purposes, despite states being heavily involved in the financing and cultural framing of mega-events. Qatar, since it gained independence from the UK in 1971, has accumulated political capital to become a sporting powerhouse in the MENA region (Middle East and North Africa). This has not come for free. Berni (65) dates a turning point to the 1994 coup d’état attempt against Qatari Emir Sheikh Hamad bin Khalifa al-Thani, allegedly staged by Saudi Arabia (KSA) and the United Arab Emirates (UAE) as payback for the Sheikh's attempts to make Qatar “an independent and self-sufficient state with a flexible foreign policy” (65). The Qataris, however, survived the coup and regrouped to carve out a new geopolitical strategy. Among the initiatives were the establishment of Al Jazeera, a global media channel where international allies could be recruited, both from within and outside the Gulf region. Some years later, disagreements over pipeline projects, the political status of groups (are they terrorists or not?) and the management of the Arab Spring, left Qatar and KSA on each side of strategically important conflicts, such as the coup in Egypt in 2013. As a result, “Qatar walks a fine line in seeking to carve out a niche of its own through its efforts to project a positive image to the world while maintaining its relations with Saudi Arabia and other Gulf states” (66).

In 2017, following a supposedly hacked email account where Qatari Emir Tamim bin Hamad Al Thani seemed to praise Hamas, Hezbollah, Iran and Israel, this balance was lost. A sea, land and air blockade by Saudi Arabia, Egypt, Bahrain, and the United Arab Emirates was rapidly implemented. Among other consequences, this blockade, which later expanded to include Jordan, Libya, and several other African countries, cut diplomatic ties and limited business opportunities. KSA's demanded that Qatar fell in line with the other Arabic countries, especially within the Gulf Cooperation Council (GCC). This was created in 1981 to integrate its member states Bahrain, Kuwait, Oman, Qatar, Saudi Arabia, and United Arab Emirates. In 2011, UAE wanted Qatar to take more responsibility as a union member in order “to counterbalance the Iranian influence in the region” (67). Demands included severing ties with terrorist organizations, shutting down Al Jazeera, and consent to scrutiny over the implementation of changes. The GCC also petitioned FIFA that a World Cup could not be hosted by a terror-funding state (68).

At first, Qatar refused to comply with any of the demands. Meanwhile, FIFA aired the idea of expanding the World Cup from 32 to 48 countries, which they had already discussed for commercial reasons (69), but, due to the lack of infrastructure in Qatar, such an expansion would require in turn an expansion of the event to its neighbouring countries. Seemingly, FIFA tried to appease the others in GCC by offering them a piece of the limelight, which was complicated by the blockade. FIFA Human Rights Policy Council, moreover, argued that moving the World Cup from one country with human rights issues to other countries with more of the same would be problematic and highlighted several weaknesses.

While the feasibility study analyzed the technical and operational infrastructure available in each of the countries, it did not include meaningful consideration of human rights risks or proposed mitigation measures, despite this being a mandatory element of FIFA's new hosting requirements (70).

Meanwhile, Qatar stood its ground and managed to reinforce national sentiment as a result of the external pressure (65). Even Russia “used shuttle diplomacy with Doha, Kuwait City, and Abu Dhabi to demonstrate Russia's commitment to promoting a resolution to the Qatar crisis and its interest in the GCC's survival as a regional organization” (65). Yet the key to it all was the US. Whereas there had been, under President Trump, a confusing inconsistency in US policy towards the Gulf crisis, the internal rivalry between American allies was seen by others in the US administration as a threat to the US presence in the region—especially given the significance of the Al-Udaid airbase and the necessity to prevent Iran's influence (71). As Iran and especially Turkey supplied Qatar with food and troops during the blockade, deepening the trench war between the blockade parties and with the World Cup coming up soon, a solution was much needed. With Trump out and Biden on his way in, and with Russia gaining traction in the region, the US upped its diplomatic efforts together with Kuwait, convincing KSA to concede its demands (72).

## The relational position

From the above, we see that the position of Qatar prior to the 2022 World Cup is relational in two ways: as part of a political world society and as part of a regional power play between GCC members. The second aspect thus concerns the relational position of an actor in a figurational network and how a sport event is exploited to improve its relative strength. This relational aspect is visible in sport as well. A reporter in the UK newspaper *Guardian* summarized a debate about soft power and the FIFA World Cup Qatar 2022™ like this:

The context here is a regional map that reads like the end of the world reinterpreted through a bloodstained Jane Austen drama. Qatar and Iran are friends. Saudi Arabia hates Iran. The United Arab Emirates hates Iran. Qatar and Saudi are pretending, for now, not to hate each other. Everyone hates Israel, apart from the US, which likes Israel while trying to maintain relations with everyone else who doesn't (74).

In other cases, the relations between actors look quite different, and the strength of a figurational approach is that it enables us to specify how the combinations are conditioned by power bargains. Elias argued that it is “impossible to understand the function A performs for B without taking into account the function B performs for A. That is what is meant when it is said that the concept of function is a concept of relationship” (39). The regional positioning of actors makes it relevant to consider Amara's (74) claim that the decision to host the 2006 Asian Games was motivated by the opportunity to portray Qatar as a regional role model for a non-Western type of modernization mentioned above, and to exploit globalization rather than protecting its citizens from the international media. In particular, the emergence of Al Jazeera played a role in rejuvenating the image of Qatar as a modern Arabic state and sporting powerhouse of the region (75).

Geopolitically, the strategic accumulation of capital through soft power efforts alone is not sufficient to survive discrediting attacks from others, which affects an actor's relational position in a network. That is why Qatar plays hardball with its gas reserves, currently the third largest in the world. They have become attractive for the European Union due to the Russian war against Ukraine and sanctions also against Europe's other main supplier, Iran. In addition, in contrast to its boycott of the 2022 Beijing Olympics, the US has been less vocal towards the 2022 FIFA World Cup in Qatar, partly because of the Al-Udeid airbase, the largest US military facility in the Middle East and the US Combat Operations Air Center for the Middle East (66). As a result, Qatar has climbed soft power rankings such as the Soft Power 30, the HEPI Soft-Power Index, and the Global Soft Power Index. In particular, the Global Soft Power Index 2021 ranks Saudi Arabia on top with Qatar coming in second. In line with Eliasian thought, this weight is relative and subject to change under certain conditions. But it is not coincidental that KSA tops the chart. According to the Index report, KSA's top score is due to more than the Saudis' improved public diplomacy, it also owes much to a long-term effort to host certain political events and alliances. Among other things relating to the Eliasian approach to power above, KSA was the first Arab state to host a G20 meeting, while also assuming “responsibility to promote and adhere to the G20 agenda, including commitment to the UN Sustainable Development Goals” (76).

Therefore, it seems as if Qatar, through the World Cup, is trying to buttress the significance of soft power through sport events by taking advantage of its vulnerability. Lacking the military might of some of its neighbours, like Iran or Saudi Arabia, soft power has been its preferred strategy from early in the 21st century. According to Amara (74), the 2006 Asian Games can be considered Qatar's breakthrough as sport event hosts. These epitomized grander societal changes both domestically and internationally. Focusing on the latter for the purposes of this article, Amara argues that Qatar's intention was to introduce a version of modernization through sport which derives from “the histories and discourses of modernity in Arabo-Islamic contexts” (*p*. 495). Since its economy is highly fossil-fuel-dependent, it has for a long time been concerned to diversify government income also, hosting international sport events as tools for generating tourism and foreign investment (77). Building upon this strategy and other sport events, Qatar has taken advantage of the momentum in gaining event experience and accumulating political capital. Since 2006, more than 600 medium to large sport events have been hosted by the country, including the Handball World Championship, the IWF Athletics World Championship, and Formula 1 Grand Prix (74), and it has also invested directly in sport through the purchase of elite clubs like football's Paris St Germain (PSG).

Outshining KSA through sport event soft power exploitations is nonetheless complicated for Qatar due to their long-term bilateral rivalry. According to Dorsey (66), since it was awarded the FIFA World Cup 2022, Qatar has had to face “organized media campaigns to undermine its credibility by the UAE as well as Israel; and an inherent reluctance by Western governments to confront head on Saudi Arabia, the largest and wealthiest of the Gulf states” (66). Even the fact that Qatar is an ally of the US did not stop KSA from lobbying heavily in the West—and especially the US—to discredit Qatar before the World Cup and raise concerns about its being a threat to regional security. And even though other states in the region finance groups that are seen by some as terrorists, the main narrative has become that the Qataris' choice of groups to finance are worse than anybody else's. Meanwhile, Qatar's attempts to portray itself as something different from its neighbours and put its mediator skills in focus, exemplified by everything from hostage situations to dialogue with the Muslim Brotherhood, reveal an incentive to study how the political capital accumulated through the FIFA World Cup Qatar 2022™ is spent in the future and how it is used in light of the country's relational position.

## The issues of scale

Lastly, the mutual relation between the stock of influence and the relational position of an actor is strengthened by the question of scale. Conceptually, soft power appears to be equally available to everybody whether it is a large authoritarian regime like China or a small liberal democracy like Norway. By contrast, this article argues that deployment of soft power in relation to sport events is connected to scale. Ontologically, scale is something other than big and small, local, or global. Social actors rely upon scale “to organize, interpret, orient, and act in their worlds are not given but made—and rather laboriously so” (78), which in effect may produce various interpretations of the significance of a country's soft power strategies and hard power tactics. For example, the battle between French and English as world language has been a soft power attempt for decades, although not described in those terms, channelled through everything from colonial empires to popular culture and political globalization debates (79). To many of those outside of these countries' colonial range, however, this battle has had lesser importance to their geopolitical opinion. Likewise, for Qatar, fans and government officials reacted differently internationally and within countries, to the significance of FIFA's ban on rainbow armbands for team captains.

Scale thus illustrates how we can address Bakalov's claim (12) that “the static “difference-in-kind categorization” of hard and soft power is quite dynamic, with a plethora of activation and feedback mechanisms” (p. 508). These nuances become relevant since the World Cup is an international event. Football fans and stakeholders all over the world claim ownership of the FIFA World Cup Qatar 2022™, even though it physically takes place in Qatar. Mega-events “serve as venues for a unique confluence of diverse actors, who are normally dispersed in time and space, allowing for an intensified interaction among individuals, ideas and infrastructures” (6). Attention to scale does not mean a collapse of levels, rather, it simply represents an incentive to explore how actors perceive the event from different sides and, in this case, how scaling is used to strengthen one's case. More specifically, the importance of Qatar as geopolitical actor has increased with the World Cup, but so has the international criticism of its human rights violations and global attention to its regional strife, which would most likely not have happened with the same force if it had not hosted the World Cup. At the same time, its quarrels with the neighbours and support from FIFA has made the event regional in a power dispute context and global as a moral discourse.

Whereas an Eliasian perspective can be criticized for not considering scaling sufficiently, Hogenstijn et al. (57) nevertheless argue that building upon, rather than dismissing, the basic interpretation of a conflict between the established and the outsiders is a fruitful field for further studies of how figuration is scaled in the case of Qatar. The reason is that although Elias and Scotson (54) did not consider it, each “geographical scale is part of a larger relational grid. It consists of vertically “stretched” and horizontally “dispersed” sociospatial processes, relations and interdependencies” (80). In practice, this means that groups can gain advantages in a conflict by a strategy of “jumping scale” (57) or can manipulate the situation where geographical scale gives them a disadvantage. Among their examples, Hogenstijn et al. (57) mention “upscaling the figuration”:

In many cases, a local figuration is connected to, or even derived from, a similar figuration on a higher spatial scale. If a group has a stronger position at higher spatial scales, it can try to use this position to increase their local power, and thus win the conflict at the local scale. Upscaling the conflict is a related strategy that is commonly used by a locally weak group (57).

This perspective applies to Qatar for several reasons. According to Chadwick, the decision by Qatar to apply for the hosting of the FIFA World Cup Qatar 2022™ was never part of any sportwashing agenda but rather an element in its geopolitical security operations. What is more, the “sudden cut from its regional partners gave Qatar further opportunity to reposition itself as a defender of peace and dialogue” (67) and strengthened the cultural exchange agreements with several European countries like Italy and France in 2018 and 2019. It also “deepened ties with “historical” partners like France and it has created or reinforced collaborations, such as with Turkey for instance” (67). In light of its rationale for hosting the World Cup against a history of regional rivalry and relation-building with hard power allies, it can be argued that Qatar used the event to scale up its geopolitical significance in several ways: to utilize the event as part of the tactics to end the blockade imposed on the country in 2017 (81), to reinforce what Elias (82) referred to as a “national habitus” or the embodied understanding of a national population's common identity, and also to dampen the criticism of its views on human rights, gender equality and LGBTQ + groups.

## Discussion and conclusion

Looking back at the conceptual history of “soft power” in 2017, Nye wrote that:

With time, I have come to realize that concepts such as soft power are like children. As an academic or a public intellectual, you can love and discipline them when they are young, but as they grow they wander off and make new company, both good and bad. There is not much you can do about it, even if you were present at the creation (22)

**Figure 1:**
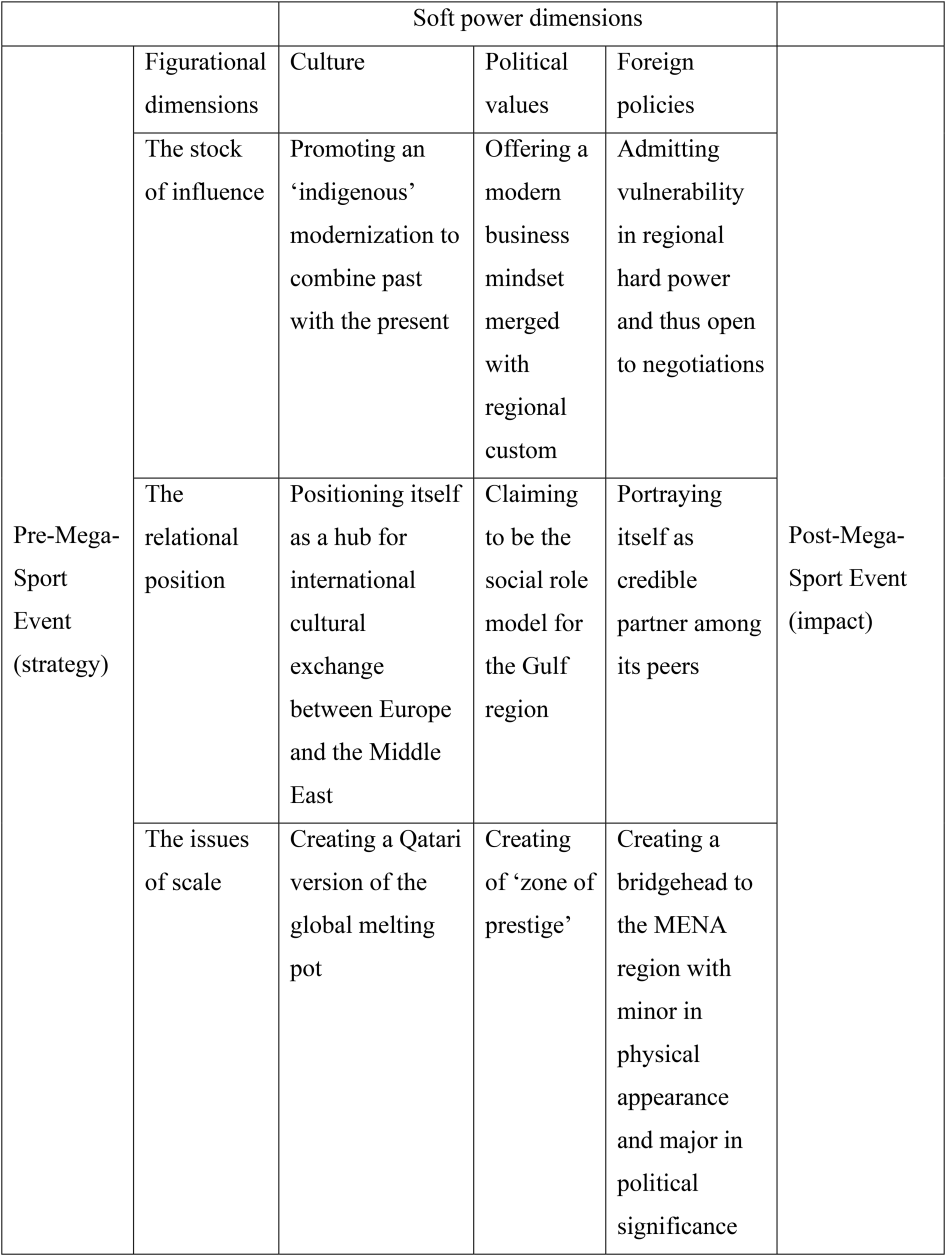
An eliasian approach to soft power and sport using the FIFA World Cup Qatar 2022™ as case.

Does this mean that some concepts are doomed to become fleeting symbols for whoever has the capacity to fill them with content? Judging from this article the answer is no, quite the contrary. As in all debates on conceptual development, the increase in usage produces the need for increased precision for analytical purposes. Drawing upon the figurational sociology of Norbert Elias, this article has argued that insight in the shifting relation between the established and the outsiders in geopolitics can improve the precision of soft power as analytical category in studies of sport. It has used the FIFA World Cup Qatar 2022™ as an example to demonstrate why history, relation and scale enrich the understanding of how states use major sporting events for soft power purposes. By coupling these geopolitical dimensions with the dimensions of soft power, Figure 1 illustrates how this framework can encourage a more holistic analysis of situations like the one in Qatar.

Starting from the left to right, the framework begins by assessing the official strategy of the event, which usually includes information about how it merges with a country's larger societal development plans. In the case of Qatar, World Cup 2022 was as much a societal project as a sporting event (77). Moving to the three figurational dimensions, the researcher is encouraged to look for evidence of each of the three in the available information about the event. When, or if, this evidence is found, which is likely in the light of earlier research on soft power and sport events, it can be analysed through the three soft power dimensions. Finally, to the right, the remaining dimensional grid (terrain which is not explored in this article) offers a point of departure to continue the analysis to include the aftermath and impact of the mega-event host's soft power tactics. Despite its holistic ambitions, the model is not intended to explain everything, neither are the dimensions exclusive to the topic of soft power and sport events. It could also be relevant to analyze other types of events which are part of a state's soft power campaigns. These events include marketing expos, academic conventions, and high-profile political summits. Apart from the fact that any model is a simplification of reality, Figure 1 also emphasizes the objective (institutional) rather than subjective (community-based) aspects of the figurational dimensions, as discussed at the end of the section “A figurational approach to power” (59).

Nonetheless, Figure 1 merges some conceptual dimensions based on Eliasian sociology which conventional soft power debates exclude in situations where the hitherto durable inequality between “established” relative to “outsider” groups in sport's “zones of prestige” (83) is challenged or altered. Underestimating the potential in soft power strategies may cause trouble for “the established group”, as Elias and Scotson underline:

 … the power to stigmatise diminishes or even goes into reverse gear when a group is no longer able to maintain its monopolisation of the principal resources of power available in a society and to exclude other interdependent groups—the former outsiders—from participation in these resources. As soon as the power disparities or, in other words, the unevenness of the balance of power, diminishes, the former outsider groups, on their part, tend to retaliate (54).

Hence, a longitudinal study of Qatar utilizing a figurational approach would most likely reveal how the World Cup affected the power dynamic between GCC actors or whether the FIFA World Cup Qatar 2022™ has borne fruit in geopolitical terms, while at the same time, it is reasonable to claim that analyses of other mega-events will identify some boxes in Figure 1 as more important to soft power aims than in the case of Qatar. As a result, the figure exemplifies how a figurational approach enables researchers to evaluate the usefulness of soft power as analytical category. Yet only an empirical analysis will reveal what the event has done for Qatar's geopolitical ambitions and the role soft power played in this process (the impact element of Figure 1).

These studies should also consider the finding that the relation between soft and hard power relative to the FIFA World Cup Qatar 2022™ confirms an image of the terms as interrelated. At the same time, hard power in this case is not domestic military force but the ability to call on stick-carrying allies when threats of armed conflict are imminent. Increased usage of “smart power” as a better term, as suggested by Nye (10), addresses this to a certain degree, but at least in connection with sport events and Qatar's relations with France after the blockade mentioned above, soft power is operationalized as cultural geopolitics. Pursuing this path further would require richer perspectives on culture and sport, as the anthropological study of soft power is underdeveloped (36). Keeping soft power as a distinct analytical category to identify mechanisms and tools through sport events is thus warranted, given theoretical additions as suggested in Figure 1, and one next step is to analyse these dimensions coherently in relation to a sporting event in order to identify the cause and effect of soft power as a strategic tool. That way, future research can specify “markers of difference” between the categories (12), as they depend on the purpose the power types are used for and how they interweave through some key dimensions of sport events. Moreover, it becomes possible to address theoretically the fluctuation of power in those situations characterized by uncontrollable dynamics, such as an arms race getting out of control or interactions based on propaganda-induced fear (48).

Lastly, although it has not been its prime concern, this article has also rejuvenated Elias' theoretical apparatus and made a claim for a figurational approach to geopolitics and sport. While there is debate on the degree to which international politics is considered by Elias (84, 50), the concept of figuration is, as demonstrated here, relevant to explore the soft power dynamics in analysis of how both small and big states use sporting events for geopolitical purposes. Earlier, as an example, it was argued that the lack of scale as analytical component in Elias and Scotson's work “hinders the analysis, as the interpretation of group behaviour at a certain scale is also dependent on the portraying of similar groups at higher spatial scales” (57). By contrast, this article has demonstrated that the basics for integrating scale in figurational analysis are there. Qatar's upscaling of the stakes in the FIFA World Cup Qatar 2022™, for instance during the blockade, created a power figuration between the adversaries and their third-party allies which would have been very different if the sport event had not been in the picture. The outcome of the use of a sport event for geopolitical purposes can thus be examined and explained by a figurational application to soft power (Figure 1), which can be used as analytical category for any type of sport event.

## Data Availability

The original contributions presented in the study are included in the article/Supplementary Materials, further inquiries can be directed to the corresponding author/s.
